# Overexpression of antibiotic resistance genes in hospital effluents over time

**DOI:** 10.1093/jac/dkx017

**Published:** 2017-02-08

**Authors:** Will P. M. Rowe, Craig Baker-Austin, David W. Verner-Jeffreys, Jim J. Ryan, Christianne Micallef, Duncan J. Maskell, Gareth P. Pearce

**Affiliations:** 1 Department of Veterinary Medicine, University of Cambridge, Cambridge, UK; 2 Institute of Integrative Biology, University of Liverpool, Liverpool, UK; 3 Centre for Environment, Fisheries and Aquaculture Science, Weymouth, UK; 4 Environment, Health and Safety, GlaxoSmithKline, Ware, UK; 5 Pharmacy Department, Addenbrooke’s Hospital, Cambridge University Hospitals NHS Foundation Trust, Cambridge, UK

## Abstract

**Objectives:** Effluents contain a diverse abundance of antibiotic resistance genes that augment the resistome of receiving aquatic environments. However, uncertainty remains regarding their temporal persistence, transcription and response to anthropogenic factors, such as antibiotic usage. We present a spatiotemporal study within a river catchment (River Cam, UK) that aims to determine the contribution of antibiotic resistance gene-containing effluents originating from sites of varying antibiotic usage to the receiving environment.

**Methods:** Gene abundance in effluents (municipal hospital and dairy farm) was compared against background samples of the receiving aquatic environment (i.e. the catchment source) to determine the resistome contribution of effluents. We used metagenomics and metatranscriptomics to correlate DNA and RNA abundance and identified differentially regulated gene transcripts.

**Results:** We found that mean antibiotic resistance gene and transcript abundances were correlated for both hospital (*ρ* = 0.9, two-tailed *P *<0.0001) and farm (*ρ* = 0.5, two-tailed *P  *<0.0001) effluents and that two β-lactam resistance genes (*bla*_GES_ and *bla*_OXA_) were overexpressed in all hospital effluent samples. High β-lactam resistance gene transcript abundance was related to hospital antibiotic usage over time and hospital effluents contained antibiotic residues.

**Conclusions:** We conclude that effluents contribute high levels of antibiotic resistance genes to the aquatic environment; these genes are expressed at significant levels and are possibly related to the level of antibiotic usage at the effluent source.

## Introduction

The rise of antibiotic resistance in clinical pathogens is occurring at an alarming rate, severely jeopardizing the sustainability of antibiotic use in human and veterinary medicine.^1^ The antibiotic resistance genes (ARGs) found in these pathogenic bacteria are thought to derive from the long-term evolution of resistance mechanisms in non-pathogenic bacteria.^2^ The resistome, comprising the resistance determinants within environmental microbial communities, offers an explanation for the diversity of ARGs that is observed in clinical situations.^3^ Indeed, there is a large body of evidence that the resistome serves as a reservoir for ARGs that can be acquired by clinically significant pathogens through transfer of mobile genetic elements (MGEs).^4^

The impact that anthropogenic activities are having on the resistome has fuelled a great deal of research and debate concerning the augmentation of the resistome and the emergence of antibiotic-resistant pathogens.^5–8^ Of particular interest is the role that effluents may have in the dissemination of ARGs and the expansion of the resistome.^9^ Using a comparative metagenomic approach, we have recently shown that effluents entering a river catchment contain ARGs and that the abundance of these ARGs is greater than that of the receiving environment, suggesting that effluents are contributing ARGs to the resistome.^10^

Metagenomics, combined with the Search Engine for Antimicrobial Resistance (SEAR),^11^ allows the resolution of full-length ARGs from environmental samples of unknown composition, thus providing an excellent method for investigating the resistome.^7^ However, it is important when assessing the impact of anthropogenic activities on the resistome that one technique is not used in isolation but rather that several techniques should be used to determine the ARG sample load.^8^ With this in mind, the identification of selection pressures and expressed ARGs within ARG-containing samples is vital information when determining the impact of anthropogenic activities on the resistome.^12^ For example, it has been found that pharmaceutical antibiotic residues present in effluents are selective pressures that can directly impact ARGs in the environment.^13^^,^^14^ However, linking selective pressures to ARG and ARG transcript abundance is not yet a feature of environmental monitoring.

In this study, we examined ARG abundance across a series of monthly effluent samples that originated from two sites of varying antibiotic usage (a municipal hospital and a dairy farm). ARG abundance was compared against background samples of the receiving aquatic environment (i.e. the source of the river catchment) to determine the relative contribution of effluents to the resistome. In addition, we employed metatranscriptomics to detect ARG transcripts and correlated them to the corresponding ARGs in each sample, facilitating the identification of differentially expressed transcripts over the sampling period. This study also assessed the selective pressures acting on the sampled microbial communities through use of LC–MS to detect antibiotic residues in the samples, as well as examining antibiotic usage data for the site of high antibiotic consumption. The combined data from this study are used to assess the impact of anthropogenic activities on the resistome.

## Materials and methods

### Sample collection and DNA and RNA sequencing

Samples were collected from three sources within the River Cam catchment, Cambridge, UK, on 2 May 2013 and five further samples over a 5 month period between August 2014 and December 2014 an average of 5 weeks apart (Table 1). Collections were made from the combined wastewater effluent of the main wards of Cambridge University Hospitals, Cambridge, UK, via a combined sewage pit (latitude 52.174343, longitude 0.139346) prior to the effluent entering the municipal sewers. Collections were also made from the effluent lagoon of the University of Cambridge dairy farm (latitude 52.22259, longitude 0.02603) and the River Cam source water (Ashwell Spring, Hertfordshire, latitude 52.0421, longitude 0.1497). The river source water served as a background sample for the environment both effluents were entering.

**Table 1:** Summary of samples used in this study

Sample	Sample type	Date collected	Latitude	Longitude	Data type	ENA accession	Total reads	Total ARG reads	% ARG reads
AH:M:1	hospital effluent	02.05.2013	52.174343	0.139346	metagenome	ERS1019923	64 659 230	97 698	0.1511
AH:M:2	hospital effluent	04.08.2014	52.174343	0.139346	metagenome	ERS1019924	52 355 416	122 164	0.2333
AH:M:3	hospital effluent	15.09.2014	52.174343	0.139346	metagenome	ERS1019925	109 795 652	207 767	0.1892
AH:M:4	hospital effluent	29.09.2014	52.174343	0.139346	metagenome	ERS1019926	61 573 380	125 019	0.203
AH:M:5	hospital effluent	27.10.2014	52.174343	0.139346	metagenome	ERS1019927	50 845 128	25 987	0.0511
AH:M:6	hospital effluent	24.11.2014	52.174343	0.139346	metagenome	ERS1019928	53 928 494	28 629	0.0531
DF:M:1	farm effluent	02.05.2013	52.22259	0.02603	metagenome	ERS1019955	66 120 642	2317	0.0035
DF:M:2	farm effluent	06.08.2014	52.22259	0.02603	metagenome	ERS1019956	184 149 408	13 094	0.0071
DF:M:3	farm effluent	15.09.2014	52.22259	0.02603	metagenome	ERS1019957	262 823 622	29 006	0.011
DF:M:4	farm effluent	29.09.2014	52.22259	0.02603	metagenome	ERS1019958	58 179 398	31 518	0.0542
DF:M:5	farm effluent	27.10.2014	52.22259	0.02603	metagenome	ERS1019959	53 192 154	6999	0.0132
DF:M:6	farm effluent	24.11.2014	52.22259	0.02603	metagenome	ERS1020022	49 5 16 248	4072	0.0082
AS:M:1	river source water	02.05.2013	52.0421	0.1497	metagenome	ERS1019949	54 799 282	181	0.0003
AS:M:2	river source water	04.08.2014	52.0421	0.1497	metagenome	ERS1019950	150 787 198	7226	0.0048
AS:M:3	river source water	15.09.2014	52.0421	0.1497	metagenome	ERS1019951	128 125 534	1199	0.0009
AS:M:4	river source water	29.09.2014	52.0421	0.1497	failed sequencing	–	–	–	–
AS:M:5	river source water	27.10.2014	52.0421	0.1497	failed sequencing	–	–	–	–
AS:M:6	river source water	24.11.2014	52.0421	0.1497	failed sequencing	–	–	–	–
AH:T:1	hospital effluent	02.05.2013	52.174343	0.139346	metatranscriptome	ERS1027345	152 298 536	308 848	0.2028
AH:T:2	hospital effluent	04.08.2014	52.174343	0.139346	failed sequencing	–	–	–	–
AH:T:3	hospital effluent	15.09.2014	52.174343	0.139346	failed sequencing	–	–	–	–
AH:T:4	hospital effluent	29.09.2014	52.174343	0.139346	metatranscriptome	ERS1027346	74 411 930	948 890	1.2752
AH:T:5	hospital effluent	27.10.2014	52.174343	0.139346	metatranscriptome	ERS1027347	61 143 518	23 765	0.0389
AH:T:6	hospital effluent	24.11.2014	52.174343	0.139346	metatranscriptome	ERS1027348	51 640 378	40 379	0.0782
DF:T:1	farm effluent	02.05.2013	52.22259	0.02603	metatranscriptome	ERS1027349	123 559 962	8017	0.0065
DF:T:2	farm effluent	04.08.2014	52.22259	0.02603	failed sequencing	–	–	–	–
DF:T:3	farm effluent	15.09.2014	52.22259	0.02603	failed sequencing	–	–	–	–
DF:T:4	farm effluent	29.09.2014	52.22259	0.02603	metatranscriptome	ERS1027350	49 293 728	4447	0.009
DF:T:5	farm effluent	27.10.2014	52.22259	0.02603	metatranscriptome	ERS1027351	64 102 402	7057	0.011
DF:T:6	farm effluent	24.11.2014	52.22259	0.02603	metatranscriptome	ERS1027352	64 850 756	1022	0.0016

AH, hospital effluent (Addenbrooke’s Hospital/Cambridge University Hospitals); DF, farm effluent (University of Cambridge dairy farm); AS, river source water (Ashwell Spring).

Samples for antibiotic residue testing were collected in 1 L sterile glass containers and transported at 4°C to the laboratory. Samples for metagenome and metatranscriptome preparation were collected in 10 L sterile polypropylene containers and transported at 4°C to the laboratory, and prokaryotic cells were isolated as described by Rowe *et al*.^10^ (2016). Each sample of prokaryotic cells was split in two, for separate DNA and RNA extractions to generate a metagenome and metatranscriptome for each sample. Metagenomes were prepared as described by Rowe *et al.*^10^ (2016). For metatranscriptome preparation, prokaryotic cells were washed in PBS solution before being treated with Max Bacterial Enhancement reagent (ThermoFisher Scientific, UK) to denature bacterial proteins and deactivate RNases. Bacterial cell lysis and RNA extraction were then performed using TRIzol reagent (ThermoFisher Scientific, UK). For each metatranscriptome, 2 μg of RNA was subjected to ribosomal RNA depletion (Ribo-Zero Gold, Epicentre, UK), quality checked using a BioAnalyzer (Agilent Technologies, US) and used to generate Illumina TruSeq RNA libraries (100 bp). All metagenome and metatranscriptome libraries were sequenced using an Illumina HiSeq2500 (Exeter Sequencing Service, UK). All sequencing data and metadata are available under the European Nucleotide Archive study with accession numbers PRJEB12083 (metagenomes) and PRJEB12284 (metatranscriptomes) (Table 1). 

### Identification of ARGs, MGEs and abundance analysis

ARGs were identified in metagenomes and metatranscriptomes using the SEAR with default parameters.^11^ MGE detection was carried out on all metagenomes as in Rowe *et al.*^10^ (2016). Briefly, reads were mapped to a custom MGE database using BWA-MEM (default options),^15^ MGEs were annotated and binned by MGE type if mapping coverage was >90%. The abundances of ARGs and MGEs were normalized to the number of 16S sequences in each metagenomic dataset, as discussed in Bengtsson-Palme *et al.*^21^ (2014). Taxonomic profiling was performed on all metagenomes using MetaPhlAn.^16^

### Antibiotic residue testing

Antibiotic residues were quantified in effluent samples using LC–MS (RPS Mountainheath, UK). Quantification standards were created for the three most used compounds in each class of antibiotics prescribed at Cambridge University Hospitals in 2013 as described below. No standards could be generated for aminoglycosides or trimethoprim.

### Antibiotic usage and statistical analysis

Monthly antibiotic usage data for periods overlapping sample collection were obtained for Cambridge University Hospitals. Hospital antibiotic usage was calculated as DDDs (per 1000 bed days), which is the assumed average maintenance dose per day for a given drug and is used as a statistical measure of drug consumption.^17^^,^^18^

Differentially regulated transcripts were identified as in Franzosa *et al.*^20^ (2014). Briefly, log DNA/RNA abundance ratios were calculated for each ARG and one-sample *t*-tests were used to determine significant deviation of abundance ratios from zero. The resulting two-tailed *P* values then underwent false discovery rate (FDR) correction using the Benjamini–Hochberg method (α = 0.05) and were used to identify differentially regulated transcripts.^19^^,^^20^

## Results

### Detection of ARGs in effluents

In order to compare the relative ARG abundance in each sample type, metagenomic DNA from three sites was sequenced on multiple occasions. This study generated 102 giga bp (Gbp) of sequencing data across all samples (Table 1). Metagenomes from three samples of the river source water failed sequencing library quality checking and were removed from the study. A total of 15 metagenomes were successfully sequenced, passed quality checking and were found to contain ARGs (Table 1). The percentage of reads matching ARGs was an average 10-fold greater in the hospital effluent samples when compared with the farm effluent samples, and ∼70-fold greater compared with the background samples of river source water (Table 1). The percentage of reads matching ARGs was an average 8-fold greater in the farm effluent compared with the background samples of river source water; however, one metagenome from a background sample was found to have a greater percentage of ARG reads than a metagenome from the farm effluent (see AS:M:2 and DF:M:1 in Table 1).

**Figure 1:**
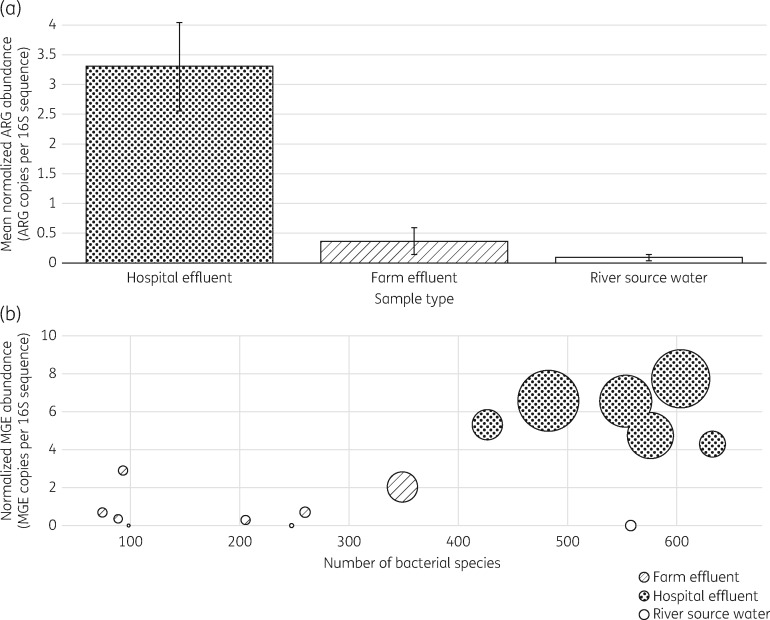
(a) Mean normalized ARG abundance across the three sample types: hospital effluent, farm effluent and background sample of river source water. The ARG abundance for each sample was normalized to the number of 16S sequences before averaging values for each sample type. Error bars depict standard errors for mean values. (b) Bubble plot showing the normalized abundance of MGEs compared with the number of bacterial species in each sample. The bubble size corresponds to the normalized ARG abundance in each sample.

After reconstructing ARGs from sequence reads using SEAR and then normalizing the ARG abundance in each sample to the 16S sequence abundance, the mean normalized abundance of ARGs in the hospital effluent samples was found to be significantly greater than in the farm effluent and background samples (9- and 34-fold greater respectively) (Figure 1a). In addition to a higher mean abundance of ARGs, the hospital effluent samples were frequently found to contain a higher abundance of MGEs and a greater number of distinct bacterial species in comparison with the farm effluent samples (Figure 1b).

### Relating ARGs in the environment at the DNA and RNA levels

In order to determine whether the ARGs that were identified in hospital and farm effluent samples (at higher abundance than those found in the background samples of river source water) were being expressed, metatranscriptomics was used to interrogate effluent samples for the presence of ARG transcripts. Two RNA samples from both the hospital and the farm effluents failed sequencing library quality checking and were removed from the study (Table 1). A total of eight metatranscriptomes were successfully sequenced, passed quality checking and were found to contain ARG transcripts at varying abundance levels (Table 1).

**Figure 2:**
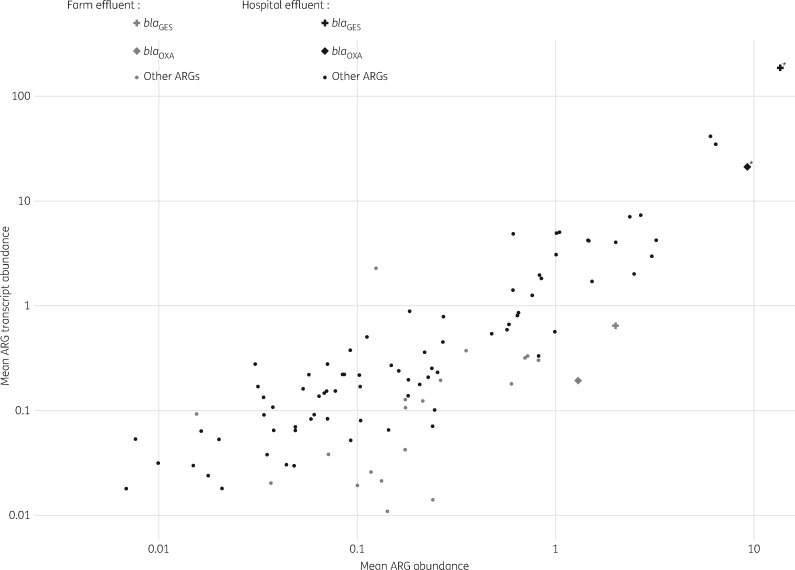
Mean ARG and ARG transcript abundance for all hospital effluent and farm effluent samples. Mean ARG and ARG transcript abundances were highly correlated for hospital (*ρ* = 0.9, two-tailed *P *<0.0001) and farm (*ρ* = 0.5, two-tailed *P *<0.0001) effluents. β-Lactam resistance genes *bla*_GES_ and *bla*_OXA_ are indicated by plus and diamond symbols, respectively. Asterisks indicate overexpressed genes (*bla*_GES_ and *bla*_OXA_) as determined by *t*-test and FDR of log DNA:RNA abundance values.

To identify differentially regulated transcripts, particularly overexpressed ARGs, across our effluent samples, we applied the global model for metagenome versus metatranscriptome regulation described by Franzosa *et al.*^20^ (2014), predicting that the mean gene and transcript abundance would be correlated across the samples if the gene regulation and rate of transcription were constant. Overall, the mean ARG and ARG transcript abundances were highly correlated for both hospital effluents (*ρ* = 0.9, two-tailed *P* <0.0001) and farm effluents (*ρ* = 0.5, two-tailed *P *<0.0001) (Figure 2). The deviation of mean log ARG/ARG transcript ratios from zero (following FDR correction) identified two differentially regulated ARG transcripts in the hospital effluent samples; the relative abundance of each of these transcripts was greater than expected (based on the abundance of the corresponding ARGs) (Figure 2). The β-lactam resistance genes *bla*_GES_ and *bla*_OXA_ were consistently overexpressed in all hospital effluent samples; a significant 11-fold mean change in transcript abundance was observed for *bla*_GES_ transcripts (two-tailed *P* <0.005, *q *<0.05) and a significant 2-fold mean change in transcript abundance was observed for *bla*_OXA_ transcripts (two-tailed *P* <0.005, *q* <0.05) [data not shown and Table S1 (available as Supplementary data at *JAC* Online)]. The β-lactam genes *bla*_GES_ and *bla*_OXA_ were present but not overexpressed in the farm effluent samples.

### Determining the effect of β-lactam antibiotic usage on ARG expression

**Figure 3:**
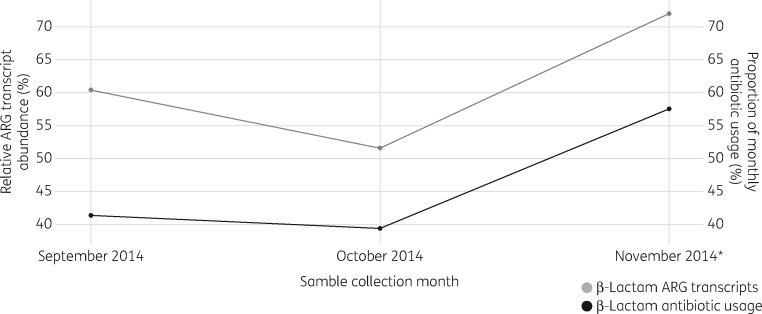
Monthly change in the relative abundance of β-lactam ARG transcripts compared with the relative β-lactam antibiotic usage for the month preceding sample collection. The asterisk indicates detection of β-lactam antibiotics in hospital effluent using LC–MS.

Following the identification of two differentially regulated ARG transcripts (*bla*_GES_ and *bla*_OXA_) in the hospital effluent samples, to investigate whether the genes were overexpressed as a result of anthropogenic activity we looked at the effect of hospital antibiotic usage on the abundance of β-lactam ARG transcripts. Antimicrobial drug usage data were obtained for the hospital and used to calculate the total volume of drugs used in the hospital each month (Table S2). Monthly drug usage for each antibiotic class was converted into a DDD (per 1000 bed days) value and the relative β-lactam usage was calculated for each month preceding metatranscriptome sample collection; monthly β-lactam usage (proportion of total usage) was found to change in line with relative ARG transcript abundance in the three sequential hospital effluent metatranscriptomes (Figure 3).

To supplement the antibiotic usage data, LC–MS was used to detect specific antibiotic residues in the effluent samples (Table S3). In total, 23 antibiotic residues from the testing panel were detected in the hospital effluent across all the sampling dates. However, in the sequentially sampled monthly metatranscriptomes, β-lactam antibiotic residues (for flucloxacillin) were only detected in November 2014 (Figure 3 and Table S3).

## Discussion

To our knowledge this work represents the first study to combine metagenomics, metatranscriptomics and an attempt to relate environmental ARG expression to antibiotic resistance selective pressures. Although there have been many good studies towards cataloguing ARG diversity in effluent-impacted waters,^21–24^ previous work in this area has been limited in terms of demonstrating how anthropogenic factors may be influencing the resistome in receiving waters. In this study we correlate, for the first time, the ARG and ARG transcripts in hospital effluents and relate this to the hospital antibiotic usage.

As previously suggested,^8^ to extend our understanding of the risks that ARGs pose to the augmentation of the resistome and the impact this may have on global health, we must go beyond the presence and absence of genes and gain a more comprehensive understanding of ARG dynamics. With this is in mind we employed metatranscriptomics to identify ARG transcripts in effluents, allowing us to elucidate the expression of key ARGs in a given sample, identify consistent overexpression across samples and classify antibiotic or ARG types that are routinely being released into the environment via effluents. Despite the complexities of generating metatranscriptomes from environmental samples,^25^ we were able to produce a sufficient number of paired metagenomes/metatranscriptomes to determine the correlation between ARG and ARG transcripts for hospital and farm effluents. Within a sample, the presence of transcripts was used as a proxy for gene expression and the ratio of ARG to ARG transcript abundance was used to determine differentially expressed transcripts.^20^ Although a commonly used method, metatranscriptomic analysis of gene expression in microbial communities may be biased by factors such as transcript half-life, causing over- or under-representation of particular genes.^26^ However, metatranscriptomics is a powerful tool as it provides a snapshot of ARG expression from a microbial community and can be linked with metagenomic data to give a taxonomic, genomic and functional overview of an environmental sample.^27^

Using the metagenomic data, we found the mean normalized ARG abundance in effluents to be much greater than in the background samples of the river catchment, particularly in the case of effluents originating from a hospital. Our results complement similar findings of ARG carriage by effluents that originate from anthropogenic sources,^28–30^ and the work described here also complements the findings of a previous study of the same river catchment by identifying additional ARG-containing effluents and sampling over an extended time period.^10^

Similar to our previous study, we encountered difficulty in obtaining sufficient biomass from several background samples, leading to failure to prepare metagenomic libraries. However, notwithstanding the reduced number of background samples, the metagenomic libraries revealed ARG abundance in hospital and farm effluents was always higher than in the corresponding background sample for the month of sampling (if available) and the mean abundance values for the effluents were significantly greater than the mean abundance value for the background river catchment.

This study also incorporated LC–MS and antibiotic usage data for the site of high antibiotic consumption, Cambridge University Hospitals. Although anecdotal, the similar trends observed between β-lactam transcript abundance and the hospital β-lactam usage over the same 3 month period, combined with the presence of antibiotics at low concentrations in the effluents, gives weight to the argument that anthropogenic activities may impact ARGs in the environment.^31^ It should be noted that the LC–MS data were for only a panel of antibiotics and the LC–MS standards used were designed for undecayed antibiotics, and consequently this LC–MS screen only provided a snapshot of selected antibiotic compounds that were present and will not have detected decayed compounds.

In terms of the overexpression of β-lactam resistance genes, the genes *bla*_GES_ and *bla*_OXA_ were expressed in both hospital and farm effluents but they were only found to be overexpressed in hospital effluents. This significant overexpression of ARGs occurring in an environment that is heavily impacted by antibiotic use, compared with the much lower expression found in farm effluents (subject to a much lower use of antibiotics), may be an example of human activity augmenting the aquatic resistome.

The combined data from this study were used to assess the impact of anthropogenic activities on the resistome. To date there have been limited temporal studies in this area and the use of background sampling is still to be routinely adopted. The powerful combination of metagenomics and metatranscriptomics provides further insight into a complex problem and we believe this to be the first report of ARGs being overexpressed in effluents. Although we try to link between ARG expression and antibiotic usage at the effluent source (Figure 3), it must be noted that there are many confounding factors (e.g. higher temperatures of effluents) that may be responsible for the observed overexpression of ARGs and further work is needed to support an association between ARG expression in effluents and antibiotic usage at the effluent source. Indeed, more must be done to investigate factors such as the genetic context of ARGs (e.g. promoter proximity) and the metabolic activity of the community being sampled, which could both impact gene expression in microbial communities. For instance, the expression of ARGs we have observed in this study could be attributed to the high temperatures of hospital effluents and the recent excretion of bacteria from human hosts. Untangling the various factors that could be involved in the observed overexpression of ARGs remains a significant challenge and determining the fate of these genes once the effluents are received by the wider aquatic environment will go towards facilitating our understanding of this complex interaction between ARGs and the environment.

Combining genomic technologies and environmental metadata to inform antibiotic stewardship is an exciting prospect and one that is being actively pursued. Future studies should consider including metatranscriptomics to identify ARG transcripts in environmental samples, developing the methodology further or using a single-cell approach to gain greater resolution of the species involved in the expression of genes of interest.^32^

While similar approaches that combine metagenomics and metatranscriptomics have been used to study the impact of anthropogenic activities on microbial communities,^33^ our study combined metagenomics, metatranscriptomics and antibiotic usage data to identify hospital effluents containing consistently overexpressed ARGs. With the suggestion of incorporating gene weighting or scoring systems into environmental antibiotic resistance risk assessments,^7^^,^^34^ the findings presented here suggest that it would be worthwhile incorporating ARG expression data when designing ARG scoring matrices in future risk assessments.

To conclude, our results indicate that effluents originating from sites of antibiotic usage and entering a river catchment are regularly contributing ARGs to the resistome; these ARGs are expressed and are more abundant than in background samples of the river catchment. The clinical impact of these ARGs re-entering the human population after release into the natural environment is clearly an area of future work, and consideration must also be given to the role of effluents in effective antibiotic stewardship.

## Supplementary Material

Supplementary Data
