# Supplementary material for: Overexpression of antibiotic resistance genes in hospital effluents over time
Source: J Antimicrob Chemother. 2017 Feb 8;72(6):1617–23. doi: 10.1093/jac/dkx017 (PMC5437528; doi:10.1093/jac/dkx017)
Supplement: Supplementary Data [file dkx017_Supp.docx]

# Supplementary data

**Table S1.** Table showing the normalised abundance values for each ARG present in the hospital effluent samples

| **ARG** | **Normalised Abundance Value** | | **Collection Date** |
| --- | --- | --- | --- |
| *(ARG-Annot annotation)* | *metagenome* | *metatranscriptome* |  |
| Aac3 | 1.001680112 | 2.240732343 | 02.05.2013 |
| Aac3 | 1.47875798 | 5.012870683 | 29.09.2014 |
| Aac3 | 0.218375733 | 0.12775842 | 27.10.2014 |
| Aac3 | 0.625396391 | 0.393728223 | 24.11.2014 |
| Aac6-Aph2 | 0.833333333 | 0.402777778 | 02.05.2013 |
| Aac6-Aph2 | 0.151388889 | 0 | 29.09.2014 |
| Aac6-Aph2 | 0 | 0 | 27.10.2014 |
| Aac6-Aph2 | 0 | 0 | 24.11.2014 |
| Aac6 | 0.163934426 | 0.544626594 | 02.05.2013 |
| Aac6 | 0 | 0 | 29.09.2014 |
| Aac6 | 0.093073593 | 0 | 27.10.2014 |
| Aac6 | 0 | 0 | 24.11.2014 |
| AadA | 2.168419765 | 4.207967344 | 02.05.2013 |
| AadA | 2.843196473 | 12.02887902 | 29.09.2014 |
| AadA | 0.12989861 | 0 | 27.10.2014 |
| AadA | 0.756313131 | 0.292929293 | 24.11.2014 |
| Ant''Ia | 1.154696133 | 3.4106814 | 02.05.2013 |
| Ant''Ia | 0.889502762 | 3.086556169 | 29.09.2014 |
| Ant''Ia | 0.624309392 | 0.241252302 | 27.10.2014 |
| Ant''Ia | 0.727440147 | 0.491712707 | 24.11.2014 |
| Ant3''Ih-Aac6-IId | 7.92887931 | 32.06896552 | 02.05.2013 |
| Ant3''Ih-Aac6-IId | 11.74137931 | 132.075431 | 29.09.2014 |
| Ant3''Ih-Aac6-IId | 2.270833333 | 0 | 27.10.2014 |
| Ant3''Ih-Aac6-IId | 2.238505747 | 0 | 24.11.2014 |
| Ant6-Ia | 0.410341034 | 0.867986799 | 02.05.2013 |
| Ant6-Ia | 0 | 0 | 29.09.2014 |
| Ant6-Ia | 0 | 0 | 27.10.2014 |
| Ant6-Ia | 0 | 0 | 24.11.2014 |
| Aph3 | 2.337814466 | 4.10804199 | 02.05.2013 |
| Aph3 | 4.819093102 | 10.83659822 | 29.09.2014 |
| Aph3 | 0.475490196 | 0.539215686 | 27.10.2014 |
| Aph3 | 0.425605808 | 0.553921569 | 24.11.2014 |
| Aph4-Ia | 0 | 0.178362573 | 02.05.2013 |
| Aph4-Ia | 0 | 0 | 29.09.2014 |
| Aph4-Ia | 0 | 0 | 27.10.2014 |
| Aph4-Ia | 0 | 0 | 24.11.2014 |
| AphA | 0.776472604 | 0.406289308 | 02.05.2013 |
| AphA | 0.960653892 | 0.588679245 | 29.09.2014 |
| AphA | 0.8197636 | 0.322012579 | 27.10.2014 |
| AphA | 0.730920392 | 0 | 24.11.2014 |
| ArmA | 0 | 0 | 02.05.2013 |
| ArmA | 0.118863049 | 0 | 29.09.2014 |
| ArmA | 0 | 0 | 27.10.2014 |
| ArmA | 0 | 0 | 24.11.2014 |
| Sat-2A | 0.401904762 | 0.824761905 | 02.05.2013 |
| Sat-2A | 0.161904762 | 0.245714286 | 29.09.2014 |
| Sat-2A | 0 | 0 | 27.10.2014 |
| Sat-2A | 0.03047619 | 0 | 24.11.2014 |
| Sat4A | 0.29281768 | 0.672191529 | 02.05.2013 |
| Sat4A | 0.097605893 | 0 | 29.09.2014 |
| Sat4A | 0 | 0 | 27.10.2014 |
| Sat4A | 0.023941068 | 0 | 24.11.2014 |
| Spc | 0.058748404 | 0.212005109 | 02.05.2013 |
| Spc | 0.021711367 | 0 | 29.09.2014 |
| Spc | 0 | 0 | 27.10.2014 |
| Spc | 0 | 0 | 24.11.2014 |
| StrA | 2.171641791 | 4.666666667 | 02.05.2013 |
| StrA | 3.84079602 | 21.14179104 | 29.09.2014 |
| StrA | 2.180348259 | 1.26119403 | 27.10.2014 |
| StrA | 1.289800995 | 1.017412935 | 24.11.2014 |
| StrB | 2.620071685 | 4.219832736 | 02.05.2013 |
| StrB | 4.224611708 | 21.96176822 | 29.09.2014 |
| StrB | 2.485065711 | 1.498207885 | 27.10.2014 |
| StrB | 1.424133811 | 1.37037037 | 24.11.2014 |
| AIM-1 | 0.049342105 | 0 | 02.05.2013 |
| AIM-1 | 0 | 0 | 29.09.2014 |
| AIM-1 | 0.04495614 | 0 | 27.10.2014 |
| AIM-1 | 0 | 0 | 24.11.2014 |
| AmpC1_Ecoli | 0 | 0.057603687 | 02.05.2013 |
| AmpC1_Ecoli | 0 | 0 | 29.09.2014 |
| AmpC1_Ecoli | 0 | 0 | 27.10.2014 |
| AmpC1_Ecoli | 0 | 0 | 24.11.2014 |
| AmpC2_Ecoli | 0.03968254 | 0.126102293 | 02.05.2013 |
| AmpC2_Ecoli | 0 | 0 | 29.09.2014 |
| AmpC2_Ecoli | 0 | 0 | 27.10.2014 |
| AmpC2_Ecoli | 0 | 0 | 24.11.2014 |
| AMPH_Ecoli | 0.040587219 | 0.151122625 | 02.05.2013 |
| AMPH_Ecoli | 0 | 0 | 29.09.2014 |
| AMPH_Ecoli | 0.100172712 | 0 | 27.10.2014 |
| AMPH_Ecoli | 0 | 0 | 24.11.2014 |
| CEPA-29 | 0 | 0 | 02.05.2013 |
| CEPA-29 | 0.032115172 | 0 | 29.09.2014 |
| CEPA-29 | 0 | 0 | 27.10.2014 |
| CEPA-29 | 0 | 0 | 24.11.2014 |
| cfxA | 0.197722567 | 0.282608696 | 02.05.2013 |
| cfxA | 0.089026915 | 0 | 29.09.2014 |
| cfxA | 0 | 0 | 27.10.2014 |
| cfxA | 0.673913043 | 0 | 24.11.2014 |
| CMY-95 | 0 | 0.212914485 | 02.05.2013 |
| CMY-95 | 0 | 0 | 29.09.2014 |
| CMY-95 | 0 | 0 | 27.10.2014 |
| CMY-95 | 0.030541012 | 0 | 24.11.2014 |
| FOX-8 | 0 | 0 | 02.05.2013 |
| FOX-8 | 0 | 0.183637946 | 29.09.2014 |
| FOX-8 | 0 | 0 | 27.10.2014 |
| FOX-8 | 0 | 0 | 24.11.2014 |
| GES | 18.52430556 | 144.2199074 | 02.05.2013 |
| GES | 33.85763889 | 576.7592593 | 29.09.2014 |
| GES | 0.149305556 | 1.203703704 | 27.10.2014 |
| GES | 1.854166667 | 18.25347222 | 24.11.2014 |
| IMP | 0.551956815 | 2.275303644 | 02.05.2013 |
| IMP | 0.183535763 | 0.102564103 | 29.09.2014 |
| IMP | 0 | 0.510121457 | 27.10.2014 |
| IMP | 0 | 0.638326586 | 24.11.2014 |
| NDM-8 | 0.146371464 | 0 | 02.05.2013 |
| NDM-8 | 0.36900369 | 0.774907749 | 29.09.2014 |
| NDM-8 | 0.184501845 | 0.351783518 | 27.10.2014 |
| NDM-8 | 0.382533825 | 0.66297663 | 24.11.2014 |
| OXA | 16.66912084 | 41.69039239 | 02.05.2013 |
| OXA | 13.25914898 | 27.27163011 | 29.09.2014 |
| OXA | 2.775142509 | 6.600065377 | 27.10.2014 |
| OXA | 4.364083598 | 8.544976706 | 24.11.2014 |
| Penicillin_Binding_Protein_Ecoli | 0 | 0.121451104 | 02.05.2013 |
| Penicillin_Binding_Protein_Ecoli | 0 | 0 | 29.09.2014 |
| Penicillin_Binding_Protein_Ecoli | 0.140904311 | 0 | 27.10.2014 |
| Penicillin_Binding_Protein_Ecoli | 0.035226078 | 0 | 24.11.2014 |
| PER-6 | 0 | 0 | 02.05.2013 |
| PER-6 | 0.040992449 | 0 | 29.09.2014 |
| PER-6 | 0 | 0 | 27.10.2014 |
| PER-6 | 0 | 0 | 24.11.2014 |
| TEM | 1.840317633 | 7.206349661 | 02.05.2013 |
| TEM | 1.63946473 | 4.242642965 | 29.09.2014 |
| TEM | 0.491289199 | 3.368176539 | 27.10.2014 |
| TEM | 0.226480836 | 5.218350755 | 24.11.2014 |
| VEB-8 | 0.083333333 | 0.072222222 | 02.05.2013 |
| VEB-8 | 0 | 0 | 29.09.2014 |
| VEB-8 | 0 | 0 | 27.10.2014 |
| VEB-8 | 0 | 0 | 24.11.2014 |
| VIM-36 | 3.5917603 | 2.993757803 | 02.05.2013 |
| VIM-36 | 0.799001248 | 2.062421973 | 29.09.2014 |
| VIM-36 | 3.948813983 | 1.802746567 | 27.10.2014 |
| VIM-36 | 1.624219725 | 1.114856429 | 24.11.2014 |
| Qnr-A1 | 0.065449011 | 0.254185693 | 02.05.2013 |
| Qnr-A1 | 0 | 0 | 29.09.2014 |
| Qnr-A1 | 0 | 0 | 27.10.2014 |
| Qnr-A1 | 0 | 0 | 24.11.2014 |
| QnrS2 | 0.395738204 | 0.537290715 | 02.05.2013 |
| QnrS2 | 1.840182648 | 1.811263318 | 29.09.2014 |
| QnrS2 | 0 | 0 | 27.10.2014 |
| QnrS2 | 0.04261796 | 0 | 24.11.2014 |
| VanA-A | 0.135658915 | 0.361434109 | 02.05.2013 |
| VanA-A | 0 | 0 | 29.09.2014 |
| VanA-A | 0 | 0 | 27.10.2014 |
| VanA-A | 0 | 0 | 24.11.2014 |
| VanB | 0.131195335 | 0.5898931 | 02.05.2013 |
| VanB | 0.08260447 | 0.0505345 | 29.09.2014 |
| VanB | 0 | 0 | 27.10.2014 |
| VanB | 0 | 0 | 24.11.2014 |
| VanH | 0.262804342 | 0.876823504 | 02.05.2013 |
| VanH | 0.074074074 | 0 | 29.09.2014 |
| VanH | 0 | 0 | 27.10.2014 |
| VanH | 0 | 0 | 24.11.2014 |
| VanR | 0.222257053 | 0.776280042 | 02.05.2013 |
| VanR | 0.122727273 | 0.098484848 | 29.09.2014 |
| VanR | 0 | 0 | 27.10.2014 |
| VanR | 0 | 0 | 24.11.2014 |
| VanS-B | 0.173363095 | 0.609375 | 02.05.2013 |
| VanS-B | 0.107142857 | 0 | 29.09.2014 |
| VanS-B | 0 | 0 | 27.10.2014 |
| VanS-B | 0 | 0 | 24.11.2014 |
| VanW-B | 0.134057971 | 0.873188406 | 02.05.2013 |
| VanW-B | 0.094202899 | 0 | 29.09.2014 |
| VanW-B | 0 | 0 | 27.10.2014 |
| VanW-B | 0 | 0 | 24.11.2014 |
| VanX | 0.279146141 | 1.495894909 | 02.05.2013 |
| VanX | 0.090311987 | 0 | 29.09.2014 |
| VanX | 0 | 0 | 27.10.2014 |
| VanX | 0 | 0 | 24.11.2014 |
| VanY | 0.362836692 | 1.939534011 | 02.05.2013 |
| VanY | 0.085501859 | 0.061957869 | 29.09.2014 |
| VanY | 0 | 0 | 27.10.2014 |
| VanY | 0 | 0 | 24.11.2014 |
| VanZ-A | 0.195473251 | 0.257201646 | 02.05.2013 |
| VanZ-A | 0 | 0 | 29.09.2014 |
| VanZ-A | 0 | 0 | 27.10.2014 |
| VanZ-A | 0 | 0 | 24.11.2014 |
| EreA | 1.095823096 | 2.889434889 | 02.05.2013 |
| EreA | 1.084357084 | 2.457821458 | 29.09.2014 |
| EreA | 0.108108108 | 0 | 27.10.2014 |
| EreA | 0.154791155 | 0.253890254 | 24.11.2014 |
| ErmA | 0.071038251 | 0.095628415 | 02.05.2013 |
| ErmA | 0 | 0 | 29.09.2014 |
| ErmA | 0 | 0 | 27.10.2014 |
| ErmA | 0 | 0 | 24.11.2014 |
| ErmB | 1.619241192 | 2.62195122 | 02.05.2013 |
| ErmB | 0.833333333 | 0.578590786 | 29.09.2014 |
| ErmB | 0 | 0 | 27.10.2014 |
| ErmB | 0.120596206 | 0 | 24.11.2014 |
| ErmF | 3.789013733 | 5.470661673 | 02.05.2013 |
| ErmF | 1.986267166 | 1.13732834 | 29.09.2014 |
| ErmF | 0.087390762 | 0.174781523 | 27.10.2014 |
| ErmF | 0.253433208 | 0 | 24.11.2014 |
| ErmG | 0.055782313 | 0 | 02.05.2013 |
| ErmG | 0.107482993 | 0 | 29.09.2014 |
| ErmG | 0 | 0 | 27.10.2014 |
| ErmG | 0 | 0 | 24.11.2014 |
| ErmT | 0.371428571 | 0.206802721 | 02.05.2013 |
| ErmT | 0 | 0 | 29.09.2014 |
| ErmT | 0 | 0 | 27.10.2014 |
| ErmT | 0 | 0 | 24.11.2014 |
| LnuB | 0.384328358 | 0.549751244 | 02.05.2013 |
| LnuB | 0.339552239 | 0 | 29.09.2014 |
| LnuB | 0 | 0 | 27.10.2014 |
| LnuB | 0 | 0 | 24.11.2014 |
| LnuC | 0.351515152 | 0.77979798 | 02.05.2013 |
| LnuC | 0.311111111 | 0 | 29.09.2014 |
| LnuC | 0 | 0 | 27.10.2014 |
| LnuC | 0.062626263 | 0 | 24.11.2014 |
| LnuF | 0 | 0 | 02.05.2013 |
| LnuF | 0.122871046 | 0 | 29.09.2014 |
| LnuF | 0 | 0 | 27.10.2014 |
| LnuF | 0 | 0 | 24.11.2014 |
| MphA | 0.774796748 | 0.813821138 | 02.05.2013 |
| MphA | 1.498373984 | 18.28373984 | 29.09.2014 |
| MphA | 0.016260163 | 0 | 27.10.2014 |
| MphA | 0.157723577 | 0.22601626 | 24.11.2014 |
| MphE | 3.989830508 | 3.536723164 | 02.05.2013 |
| MphE | 6.094915254 | 6.934463277 | 29.09.2014 |
| MphE | 0.629378531 | 0.740112994 | 27.10.2014 |
| MphE | 1.515254237 | 0.6 | 24.11.2014 |
| MsrD | 0.157786885 | 0.364071038 | 02.05.2013 |
| MsrD | 0.085382514 | 0 | 29.09.2014 |
| MsrD | 0 | 0 | 27.10.2014 |
| MsrD | 0 | 0 | 24.11.2014 |
| MsrE | 3.769647696 | 6.305555556 | 02.05.2013 |
| MsrE | 6.084688347 | 8.289295393 | 29.09.2014 |
| MsrE | 0.99796748 | 1.275067751 | 27.10.2014 |
| MsrE | 2.019647696 | 0.907859079 | 24.11.2014 |
| VatE | 0.150387597 | 0.427906977 | 02.05.2013 |
| VatE | 0 | 0 | 29.09.2014 |
| VatE | 0 | 0 | 27.10.2014 |
| VatE | 0 | 0 | 24.11.2014 |
| CatA1 | 0.136363636 | 1.101515152 | 02.05.2013 |
| CatA1 | 0.146969697 | 0 | 29.09.2014 |
| CatA1 | 0 | 0 | 27.10.2014 |
| CatA1 | 0 | 0 | 24.11.2014 |
| CatB2 | 0.404494382 | 1.428838951 | 02.05.2013 |
| CatB2 | 0.578651685 | 1.361423221 | 29.09.2014 |
| CatB2 | 0 | 0 | 27.10.2014 |
| CatB2 | 0.106741573 | 0.342696629 | 24.11.2014 |
| CatB4 | 1.336976321 | 2.714025501 | 02.05.2013 |
| CatB4 | 4.304189435 | 13.39526412 | 29.09.2014 |
| CatB4 | 0 | 0 | 27.10.2014 |
| CatB4 | 0.18579235 | 0.599271403 | 24.11.2014 |
| FloR | 0 | 0 | 02.05.2013 |
| FloR | 0.023045267 | 0 | 29.09.2014 |
| FloR | 0 | 0 | 27.10.2014 |
| FloR | 0.023045267 | 0 | 24.11.2014 |
| PheCmlA5 | 0.066666667 | 0.173809524 | 02.05.2013 |
| PheCmlA5 | 0.128571429 | 0.103968254 | 29.09.2014 |
| PheCmlA5 | 0 | 0 | 27.10.2014 |
| PheCmlA5 | 0 | 0 | 24.11.2014 |
| Arr6 | 1.046357616 | 2.185430464 | 02.05.2013 |
| Arr6 | 2.777041943 | 9.604856512 | 29.09.2014 |
| Arr6 | 0.035320088 | 0 | 27.10.2014 |
| Arr6 | 0.17218543 | 0.432671082 | 24.11.2014 |
| SulI | 8.84047619 | 16.3 | 02.05.2013 |
| SulI | 7.04047619 | 113.6988095 | 29.09.2014 |
| SulI | 5.578571429 | 4.902380952 | 27.10.2014 |
| SulI | 4.202380952 | 3.746428571 | 24.11.2014 |
| SulII | 0.704656863 | 2.117647059 | 02.05.2013 |
| SulII | 0.784313725 | 2.18872549 | 29.09.2014 |
| SulII | 1.101715686 | 0.381127451 | 27.10.2014 |
| SulII | 0.462009804 | 0.317401961 | 24.11.2014 |
| Tet-32 | 0.1203125 | 0.319791667 | 02.05.2013 |
| Tet-32 | 0.140104167 | 0 | 29.09.2014 |
| Tet-32 | 0.094791667 | 0 | 27.10.2014 |
| Tet-32 | 0.061458333 | 0 | 24.11.2014 |
| Tet-36 | 0 | 0 | 02.05.2013 |
| Tet-36 | 0.147685907 | 0 | 29.09.2014 |
| Tet-36 | 0 | 0 | 27.10.2014 |
| Tet-36 | 0 | 0 | 24.11.2014 |
| Tet-39 | 0.441077441 | 1.054713805 | 02.05.2013 |
| Tet-39 | 2.25 | 0.97979798 | 29.09.2014 |
| Tet-39 | 0.334175084 | 0.205387205 | 27.10.2014 |
| Tet-39 | 0.938552189 | 0 | 24.11.2014 |
| Tet-40 | 0.062244062 | 0.33005733 | 02.05.2013 |
| Tet-40 | 0.061425061 | 0 | 29.09.2014 |
| Tet-40 | 0 | 0 | 27.10.2014 |
| Tet-40 | 0.110565111 | 0 | 24.11.2014 |
| Tet-44 | 0 | 0 | 02.05.2013 |
| Tet-44 | 0.05200208 | 0 | 29.09.2014 |
| Tet-44 | 0 | 0 | 27.10.2014 |
| Tet-44 | 0 | 0 | 24.11.2014 |
| TetA | 0.299607843 | 0.315294118 | 02.05.2013 |
| TetA | 0.201568627 | 0.388235294 | 29.09.2014 |
| TetA | 0.190588235 | 0 | 27.10.2014 |
| TetA | 0.135686275 | 0 | 24.11.2014 |
| TetB | 0.066334992 | 0.118573798 | 02.05.2013 |
| TetB | 0.092868988 | 0 | 29.09.2014 |
| TetB | 0 | 0 | 27.10.2014 |
| TetB | 0.033167496 | 0 | 24.11.2014 |
| TetC | 0.209907641 | 0.332493703 | 02.05.2013 |
| TetC | 0.07388749 | 0 | 29.09.2014 |
| TetC | 0 | 0 | 27.10.2014 |
| TetC | 0 | 0 | 24.11.2014 |
| TetD | 0.557805907 | 0.514767932 | 02.05.2013 |
| TetD | 0.394936709 | 0.490295359 | 29.09.2014 |
| TetD | 0 | 0 | 27.10.2014 |
| TetD | 0 | 0 | 24.11.2014 |
| TetE | 0 | 0 | 02.05.2013 |
| TetE | 0.125615764 | 0 | 29.09.2014 |
| TetE | 0 | 0 | 27.10.2014 |
| TetE | 0 | 0 | 24.11.2014 |
| TetL | 0.310820625 | 0.611474219 | 02.05.2013 |
| TetL | 0 | 0 | 29.09.2014 |
| TetL | 0 | 0 | 27.10.2014 |
| TetL | 0 | 0 | 24.11.2014 |
| TetM | 0.459895833 | 0.949479167 | 02.05.2013 |
| TetM | 0.161458333 | 0 | 29.09.2014 |
| TetM | 0 | 0 | 27.10.2014 |
| TetM | 0.027083333 | 0 | 24.11.2014 |
| TetO | 0.606770833 | 1.672916667 | 02.05.2013 |
| TetO | 0.713020833 | 0.307291667 | 29.09.2014 |
| TetO | 0.330729167 | 0.169791667 | 27.10.2014 |
| TetO | 0.2546875 | 0 | 24.11.2014 |
| TetQ | 0.131879543 | 0.260124611 | 02.05.2013 |
| TetQ | 0.244029076 | 0 | 29.09.2014 |
| TetQ | 0 | 0 | 27.10.2014 |
| TetQ | 0.199896158 | 0 | 24.11.2014 |
| TetR | 0.32718894 | 0.207373272 | 02.05.2013 |
| TetR | 0.23655914 | 0.620583717 | 29.09.2014 |
| TetR | 0.221198157 | 0 | 27.10.2014 |
| TetR | 0.129032258 | 0 | 24.11.2014 |
| TetS | 0.059709242 | 0.119418484 | 02.05.2013 |
| TetS | 0 | 0 | 29.09.2014 |
| TetS | 0 | 0 | 27.10.2014 |
| TetS | 0 | 0 | 24.11.2014 |
| TetU | 0.122641509 | 0.371069182 | 02.05.2013 |
| TetU | 0 | 0.452830189 | 29.09.2014 |
| TetU | 0 | 0 | 27.10.2014 |
| TetU | 0 | 0.27672956 | 24.11.2014 |
| TetW | 0.948958333 | 1.632291667 | 02.05.2013 |
| TetW | 0.613020833 | 1.125 | 29.09.2014 |
| TetW | 0.608333333 | 0.653645833 | 27.10.2014 |
| TetW | 0.431770833 | 0 | 24.11.2014 |
| TetX | 1.017994859 | 1.923736075 | 02.05.2013 |
| TetX | 1.11311054 | 0.71293916 | 29.09.2014 |
| TetX | 0.071122536 | 0 | 27.10.2014 |
| TetX | 0.128534704 | 0 | 24.11.2014 |
| Dfr24 | 0.023297491 | 0 | 02.05.2013 |
| Dfr24 | 0 | 0 | 29.09.2014 |
| Dfr24 | 0 | 0 | 27.10.2014 |
| Dfr24 | 0 | 0 | 24.11.2014 |
| DfrA1 | 0.5907173 | 0.609704641 | 02.05.2013 |
| DfrA1 | 0.388185654 | 0.310126582 | 29.09.2014 |
| DfrA1 | 0 | 0 | 27.10.2014 |
| DfrA1 | 0.040084388 | 0 | 24.11.2014 |
| DfrA12 | 1.034136546 | 3.497991968 | 02.05.2013 |
| DfrA12 | 2.837349398 | 14.25903614 | 29.09.2014 |
| DfrA12 | 0 | 0 | 27.10.2014 |
| DfrA12 | 0.176706827 | 1.815261044 | 24.11.2014 |
| DfrA14 | 0.113924051 | 0.215189873 | 02.05.2013 |
| DfrA14 | 0 | 0.042194093 | 29.09.2014 |
| DfrA14 | 0 | 0 | 27.10.2014 |
| DfrA14 | 0.037974684 | 0 | 24.11.2014 |
| DfrA17 | 0.126582278 | 0.672995781 | 02.05.2013 |
| DfrA17 | 0 | 0 | 29.09.2014 |
| DfrA17 | 0 | 0 | 27.10.2014 |
| DfrA17 | 0 | 0 | 24.11.2014 |
| DfrA27 | 0.02742616 | 0.071729958 | 02.05.2013 |
| DfrA27 | 0 | 0 | 29.09.2014 |
| DfrA27 | 0 | 0 | 27.10.2014 |
| DfrA27 | 0 | 0 | 24.11.2014 |
| DfrA5 | 0.550632911 | 0.723628692 | 02.05.2013 |
| DfrA5 | 0.124472574 | 0.206751055 | 29.09.2014 |
| DfrA5 | 0.109704641 | 0.5 | 27.10.2014 |
| DfrA5 | 0.0907173 | 0 | 24.11.2014 |
| DfrA8 | 0 | 0.244725738 | 02.05.2013 |
| DfrA8 | 0 | 0 | 29.09.2014 |
| DfrA8 | 0 | 0 | 27.10.2014 |
| DfrA8 | 0 | 0 | 24.11.2014 |
| DfrB2 | 0.067510549 | 0.459915612 | 02.05.2013 |
| DfrB2 | 0.042194093 | 0.071729958 | 29.09.2014 |
| DfrB2 | 0.025316456 | 0 | 27.10.2014 |
| DfrB2 | 0 | 0 | 24.11.2014 |
| DfrG | 0.273092369 | 0.582329317 | 02.05.2013 |
| DfrG | 0 | 0 | 29.09.2014 |
| DfrG | 0 | 0 | 27.10.2014 |
| DfrG | 0 | 0 | 24.11.2014 |

**Table S2.** Table showing antibiotic usage at Cambridge University Hospitals for the months of sample collection

| **Antimicrobial drug description and summary** | **Quantity used per month** | |  |  |
| --- | --- | --- | --- | --- |
|  | *Apr-13* | *Aug-14* | *Sep-14* | *Oct-14** |
| AMIKACIN 100mg/2mL INJECTION (5 vials) | 100 | 10 | 6 |  |
| AMIKACIN Injection 500mg/2ml vial (5) | 311 | 334 | 248 |  |
| AMOXICILLIN Capsules 250mg (21) | 784 | 616 | 336 |  |
| AMOXICILLIN Capsules 250mg tto A.H. (21) | 9 | 7 | 2 |  |
| AMOXICILLIN Capsules 500mg (21) | 490 | 504 | 695 |  |
| AMOXICILLIN Capsules 500mg tto (21) | 37 | 32 | 25 |  |
| AMOXICILLIN Injection 1g (10) | 60 | 390 | 110 |  |
| AMOXICILLIN Injection 250mg (10) |  | 30 | 10 |  |
| AMOXICILLIN Injection 500mg (10) | 150 | 80 | 40 |  |
| AMOXICILLIN Sachets 3g (2) |  |  | 2 |  |
| AMOXICILLIN Syrup 125mg/5ml (100ml) | 600 | 700 | 600 |  |
| AMOXICILLIN Syrup 125mg/5ml T.T.O. (100ml) | 11 | 2 | 3 |  |
| AMOXICILLIN Syrup 250mg/5ml (100ml) | 100 | 700 | 900 |  |
| AMOXICILLIN Syrup 250mg/5ml T.T.O. (100ml) | 8 | 1 | 6 |  |
| AMPICILLIN Capsules 250mg (28) | 84 |  |  |  |
| AMPICILLIN Syrup 125mg/5ml (100ml) | 100 |  |  |  |
| AZITHROMYCIN capsules 250mg (4) | 568 | 505 | 540 |  |
| AZITHROMYCIN Capsules 250mg tto (4) | 2 | 5 | 24 |  |
| AZITHROMYCIN Suspension 200mg in 5ml (30ml) | 210 | 660 | 600 |  |
| AZITHROMYCIN Tablets 250mg (4) | 104 | 144 | 248 |  |
| AZTREONAM Injection 1g (1) | 2 | 33 | 42 |  |
| AZTREONAM Injection 2g (1) | 28 | 37 | 47 |  |
| BENZATHINE BENZYLPENICILLIN Injection 1.2mu (1) | 1 |  |  |  |
| BENZATHINE BENZYLPENICILLIN Injection 2.4mu (1) |  | 9 |  |  |
| BENZYLPENICILLIN Injection 1200mg (25) | 175 | 641 | 275 |  |
| BENZYLPENICILLIN Injection 600mg (25) | 875 | 775 | 750 |  |
| CAPREOMYCIN Injection 1g (1) | 35 |  |  |  |
| CEFACLOR Suspension 125mg/5ml (100ml) |  | 200 |  |  |
| CEFALEXIN Capsules 250mg (28) | 91 | 152 | 147 |  |
| CEFALEXIN Capsules 250mg tto (28) | 8 | 11 | 5 |  |
| CEFALEXIN Capsules 500mg (21) | 413 | 231 | 185 |  |
| CEFALEXIN Capsules 500mg tto (21) | 12 | 8 | 11 |  |
| CEFALEXIN Suspension 125mg/5ml (100ml) | 500 | 600 | 600 |  |
| CEFALEXIN Suspension 250mg/5ml (100ml) | 300 | 400 | 200 |  |
| CEFOTAXIME 500mg INJECTION (1 vial) | 204 |  |  |  |
| CEFOTAXIME Injection 1g (10) | 28 | 10 | 10 |  |
| CEFOTAXIME Injection 2g (1) | 2 | 10 |  |  |
| CEFOTAXIME Injection 500mg (10) |  | 230 | 120 |  |
| CEFOXITIN 1g INJECTION (25vials) |  | 150 | 25 |  |
| CEFOXITIN 2g INJECTION (10 vials) |  |  | 10 |  |
| CEFTAZIDIME 1g INJECTION (5 vials) |  | 114 | 190 |  |
| CEFTAZIDIME 2g INJECTION (5 vials) |  | 15 | 125 |  |
| CEFTAZIDIME Injection 1g (1) | 67 |  |  |  |
| CEFTAZIDIME Injection 2g (1) | 173 |  | 0 |  |
| CEFTAZIDIME Injection 500mg (1) | 2 | 46 | 35 |  |
| CEFTRIAXONE 1g INJECTION (5 vials) | 525 | 439 | 500 |  |
| CEFTRIAXONE Injection 2g (1) | 417 | 291 | 263 |  |
| CEFUROXIME 250mg INJECTION (10 vial) | 100 | 112 | 120 |  |
| CEFUROXIME 50mg INJECTION (10 vials) |  | 220 | 140 |  |
| CEFUROXIME 750mg INJECTION (5 vials) |  | 10 |  |  |
| CEFUROXIME INTRACAMERAL INJECTION 1mg/0.1ml(1) | 155 |  |  |  |
| CEFUROXIME Tablets 250mg (14) | 7 |  |  |  |
| CHLORAMPHENICOL Injection 1g/10ml (1) | 36 | 48 | 2 |  |
| CIPROFLOXACIN Injection 100mg/50ml (1) | 4 | 3 | 5 |  |
| CIPROFLOXACIN Injection 200mg/100ml (10) | 83 |  |  |  |
| CIPROFLOXACIN Injection 200mg/100ml FLEXBAG (1) | 443 | 640 | 449 |  |
| CIPROFLOXACIN Injection 400mg/200ml (5) | 128 |  |  |  |
| CIPROFLOXACIN Injection 400mg/200ml FLEXBAG (1) | 6 | 280 | 372 |  |
| CIPROFLOXACIN Suspension 250mg/5ml (100ml) | 1800 | 2700 | 3000 |  |
| CIPROFLOXACIN Tablets 250mg (10) | 168 | 240 | 300 |  |
| CIPROFLOXACIN Tablets 250mg (20) | 1002 | 848 | 1151 |  |
| CIPROFLOXACIN Tablets 250mg (TTO) (20) | 94 |  |  |  |
| CIPROFLOXACIN Tablets 500mg (10) | 929 | 573 | 868 |  |
| CIPROFLOXACIN Tablets 500mg (20) | 2267 | 2081 | 2172 |  |
| CIPROFLOXACIN Tablets 500mg tto (10) | 141 | 223 | 202 |  |
| CLARITHROMYCIN Injection 500mg (1) | 533 | 457 | 376 |  |
| CLARITHROMYCIN Suspension 125mg/5ml (70ml) | 770 | 140 | 1050 |  |
| CLARITHROMYCIN Suspension 125mg/5ml T.T.O. (100ml) | 8 | 6 | 10 |  |
| CLARITHROMYCIN Suspension 250mg/5ml (70ml) | 1680 | 1820 | 1330 |  |
| CLARITHROMYCIN Tablets 250mg (14) | 978 | 759 | 674 |  |
| CLARITHROMYCIN Tablets 250mg TTO (14) | 16 | 2 | 23 |  |
| CLARITHROMYCIN Tablets 500mg (14) | 2415 | 557 | 767 |  |
| CLARITHROMYCIN Tablets 500mg tto (14) | 64 | 100 | 123 |  |
| CLINDAMYCIN Capsules 150mg (100) | 569 | 1497 | 407 |  |
| CLINDAMYCIN Injection 150mg/ml (2ml) (5) | 161 | 160 | 40 |  |
| CLINDAMYCIN Injection 150mg/ml 4ml (5) | 251 | 242 | 180 |  |
| CLINDAMYCIN Suspension 75mg/5ml (80ml) |  | 320 |  |  |
| CLOFAZIMINE 100mg CAPSULES (100 caps) | 21 | 30 |  |  |
| CO-AMOXICLAV Injection 1.2G (10) | 5971 | 5570 | 5200 |  |
| CO-AMOXICLAV Injection 600mg (10) | 80 | 110 | 100 |  |
| CO-AMOXICLAV Suspension 125/31 in 5ml (100ml) | 2800 | 2400 | 2500 |  |
| CO-AMOXICLAV Suspension 125/31 in 5ml T.T.O. (100ml) | 26 | 27 | 44 |  |
| CO-AMOXICLAV Suspension 250/62 in 5ml (100ml) | 12800 | 14800 | 13900 |  |
| CO-AMOXICLAV Suspension 250/62 in 5ml T.T.O. (100ml) | 23 | 21 |  |  |
| CO-AMOXICLAV Tablets 375mg (21) | 1055 | 505 | 117 |  |
| CO-AMOXICLAV Tablets 375mg tto (21) | 53 | 52 | 36 |  |
| CO-AMOXICLAV Tablets 625mg (21) | 10732 | 4049 | 3385 |  |
| CO-AMOXICLAV Tablets 625mg tto (21) | 612 | 928 | 979 |  |
| COLISTIN Injection 1,000,000u (10) | 134 | 121 | 40 |  |
| COLISTIN NEBULISING SOLUTION Powder 80mg (30) |  | 30 | 30 |  |
| CO-TRIMOXAZOLE 480mg/5mL INJECTION (5 amps) |  | 411 | 42 |  |
| CO-TRIMOXAZOLE Injection 480mg/5ml (10) | 701 | 15 | 764 |  |
| CO-TRIMOXAZOLE Suspension 240mg/5ml (100ml) | 2800 | 3350 | 3000 |  |
| CO-TRIMOXAZOLE Suspension 480mg/5ml (100ml) | 2900 | 1900 | 1500 |  |
| CO-TRIMOXAZOLE Tablets 480mg (100) | 1348 | 1596 | 1006 |  |
| CO-TRIMOXAZOLE Tablets 480mg (28) | 112 | 196 | 224 |  |
| CO-TRIMOXAZOLE Tablets 960mg (100) | 518 | 722 | 622 |  |
| CYCLOSERINE Capsules 250mg (100) | 100 | 72 | 30 |  |
| DAPSONE Tablets 100mg (28) | 6 | 12 | 28 |  |
| DAPSONE Tablets 50mg (28) | 8 |  |  |  |
| DAPTOMYCIN 350mg VIAL (1) | 17 |  | 8 |  |
| DAPTOMYCIN 500mg INJECTION (1 vial) | 9 | 28 | 15 |  |
| DEMECLOCYCLINE Capsules 150mg (28) | 182 | 155 | 100 |  |
| DOXYCYCLINE Capsules 100mg (50) | 1142 | 1136 | 1903 |  |
| DOXYCYCLINE Capsules 100mg (8) | 8 | 8 | 48 |  |
| DOXYCYCLINE Capsules 100mg tto (14) | 47 | 55 | 87 |  |
| DOXYCYCLINE Capsules 50mg (28) | 7 | 28 |  |  |
| DOXYCYCLINE Dispersible tablets 100mg (8) | 18 | 8 | 25 |  |
| ERTAPENEM Injection 1g (1) | 181 | 187 | 114 |  |
| ERYTHROMYCIN Injection 1gram (1) | 19 | 42 | 62 |  |
| ERYTHROMYCIN Suspension 125mg/5ml (100ml) | 100 | 300 | 500 |  |
| ERYTHROMYCIN Suspension 250mg/5ml (100ml) | 800 | 700 | 900 |  |
| ERYTHROMYCIN Suspension 250mg/5ml t.t.o. (100ml) |  | 2 | 1 |  |
| ERYTHROMYCIN Tablets 250mg (28) | 284 | 32 | 476 |  |
| ERYTHROMYCIN Tablets 250mg tto (28) | 17 | 20 | 9 |  |
| ETHAMBUTOL HYDROCHLORIDE Tablets 100mg (56) | 56 | 388 | 285 |  |
| ETHAMBUTOL HYDROCHLORIDE Tablets 400mg (56) | 56 | 504 | 168 |  |
| FLUCLOXACILLIN Capsules 250mg (28) | 392 | 336 | 476 |  |
| FLUCLOXACILLIN Capsules 250mg tto (28) | 3 | 10 | 8 |  |
| FLUCLOXACILLIN Capsules 500mg (28) | 2520 | 2487 | 2780 |  |
| FLUCLOXACILLIN Capsules 500mg tto (28) | 98 | 103 | 97 |  |
| FLUCLOXACILLIN Injection 1G (10) | 2364 | 2320 | 1901 |  |
| FLUCLOXACILLIN Injection 250mg (10) | 300 | 90 | 250 |  |
| FLUCLOXACILLIN Injection 500mg (10) | 310 | 200 | 270 |  |
| FLUCLOXACILLIN Syrup 125mg/5ml (100ml) | 1200 | 1000 | 400 |  |
| FLUCLOXACILLIN Syrup 125mg/5ml T.T.O. (100ml) | 9 | 12 | 8 |  |
| FLUCLOXACILLIN Syrup 250mg/5ml (100ml) | 700 | 1800 | 1400 |  |
| FLUCLOXACILLIN Syrup 250mg/5ml T.T.O. (100ml) | 4 |  |  |  |
| FOSFOMYCIN 3g SACHETS (1) | 5 | 1 | 1 |  |
| GENTAMICIN 20mg/2ml INJECTION (5 x 2mL amps) |  | 485 | 490 |  |
| GENTAMICIN 80mg/2mL INJECTION (10 amps) | 2722 | 2920 | 3231 |  |
| GENTAMICIN Chains (1) | 3 | 4 | 4 |  |
| GENTAMICIN Injection 20mg/2ml (5) | 405 |  |  |  |
| GENTAMICIN Intrathecal injection 5mg/1ml (5) | 10 | 20 | 35 |  |
| IMIPENEM 500mg & CILASTATIN 500mg INFUSION (10 vials) |  | 255 | 20 |  |
| ISONIAZID 50mg/2mL INJECTION (10 amps) | 170 | 30 |  |  |
| ISONIAZID Solution 50mg/5ml (500ml) |  |  | 500 |  |
| ISONIAZID Tablets 100mg (28) | 224 | 560 | 308 |  |
| LEVOFLOXACIN 500mg TABLETS (5 tabs) | 65 |  |  |  |
| LINEZOLID Capsules 600mg (10) | 101 | 231 | 219 |  |
| LINEZOLID Injection 600mg (300ml) | 171 | 346 | 308 |  |
| LINEZOLID Suspension 20mg/ml (150ml) |  | 2550 | 900 |  |
| LYMECYCLINE Capsules 408MG (28) |  | 14 | 28 |  |
| MEROPENEM Injection 1g (10) | 2450 | 2861 | 2125 |  |
| MEROPENEM Injection 500mg (10) | 4098 | 4140 | 4181 |  |
| METRONIDAZOLE 500mg/100mL INFUSION (20 bags) | 1307 | 1757 | 1166 |  |
| METRONIDAZOLE Suppositories 1g (10) | 50 | 30 | 60 |  |
| METRONIDAZOLE Suppositories 500mg (10) | 20 | 10 |  |  |
| METRONIDAZOLE Suspension 200mg/5ml (100ml) | 2700 | 1200 | 1700 |  |
| METRONIDAZOLE Tablets 200mg (21) | 203 | 200 | 63 |  |
| METRONIDAZOLE Tablets 400mg (21) | 1606 | 1188 | 1599 |  |
| METRONIDAZOLE Tablets 400mg tto (21) | 124 | 111 | 121 |  |
| MINOCYCLINE Tablets 100mg (28) |  | 84 | 28 |  |
| MOXIFLOXACIN 400mg/250mL INFUSION | 67 |  |  |  |
| MOXIFLOXACIN 400mg/250ml INFUSION (1 btl) |  | 4 | 1 |  |
| MOXIFLOXACIN Tablets 400mg (5) | 103 | 206 | 133 |  |
| NITROFURANTOIN 50mg CAPSULES (TTO) (30 caps) |  | 2 |  |  |
| NITROFURANTOIN Capsules 50mg (30) | 410 | 306 | 482 |  |
| NITROFURANTOIN Tablets 100mg (28) | 308 | 208 | 125 |  |
| NORFLOXACIN Tablets 400mg (14) | 70 |  |  |  |
| OFLOXACIN Tablets 200mg (10) | 120 | 30 | 110 |  |
| OFLOXACIN Tablets 200mg tto (10) | 10 | 10 | 10 |  |
| OFLOXACIN Tablets 400mg tto (10) | 25 | 13 | 16 |  |
| OXYTETRACYCLINE Tablets 250mg (28) |  | 140 | 56 |  |
| PENICILLIN V Elixir 125mg/5ml (100ml) | 400 | 400 | 100 |  |
| PENICILLIN V Elixir 125mg/5ml t.t.o. (100ml) | 9 | 2 | 6 |  |
| PENICILLIN V Elixir 250mg/5ml (100ml) | 1000 | 1100 | 800 |  |
| PENICILLIN V Elixir 250mg/5ml tto (100ml) | 12 | 4 | 1 |  |
| PENICILLIN V Tablets 250mg (28) | 1204 | 972 | 1356 |  |
| PENICILLIN V Tablets 250mg tto (28) | 88 | 64 | 46 |  |
| PIPERACILLIN 2g TAZOBACTAM 0.25g INJ (Fresenius) (10) |  | 174 | 220 |  |
| PIPERACILLIN 4g TAZOBACTAM 0.5g INJ (10) |  | 6638 | 6898 |  |
| PIVMECILLINAM 200mg Tablets (10) |  | 30 |  |  |
| PRIMAXIN 500mg (Imipenem) INFUSION (1 vial) | 171 |  |  |  |
| Procaine Benzylpenicillin 1.2mu in 5ml Injection (1vial) |  |  | 20 |  |
| PROTIONAMIDE Tablets 250mg (50) | 50 | 114 | 136 |  |
| PYRAZINAMIDE 500mg TABLETS (30 tabs) |  | 240 | 270 |  |
| PYRAZINAMIDE 500mg TABLETS (60 tabs) | 180 |  |  |  |
| PYRIMETHAMINE Tablets 25mg (30) | 40 | 60 |  |  |
| RIFABUTIN Capsules 150mg (30) |  | 90 | 90 |  |
| RIFAMPICIN Capsules 150mg (100) | 0 | 44 |  |  |
| RIFAMPICIN Capsules 300mg (100) | 610 | 207 | 92 |  |
| RIFAMPICIN Injection 600mg (1) | 73 | 35 | 34 |  |
| RIFAMPICIN Syrup 100mg/5ml (120ml) | 360 | 120 | 120 |  |
| RIFATER Tablets (100) | 200 | 418 | 161 |  |
| RIFAXIMIN 200mg TABLETS (9 tabs) | 306 |  |  |  |
| RIFAXIMIN 550mg TABLETS (56 tabs) | 448 | 482 | 420 |  |
| RIFINAH Tablets (56) |  | 56 | 56 |  |
| SOFOSBUVIR 400mg LEDIPASVIR 90mg TABLET (30 tabs) |  | 60 |  |  |
| SPIRAMYCIN Tablets 500mg (16) |  |  | 272 |  |
| SULFADIAZINE Tablets 500mg (56) | 84 | 112 |  |  |
| TAZOCIN Injection 2.25g (1) | 246 |  |  |  |
| TAZOCIN Injection 4.5g (1) | 7103 |  |  |  |
| TEICOPLANIN Injection 200mg (1) | 186 | 112 | 158 |  |
| TEICOPLANIN Injection 400mg (1) | 150 | 187 | 200 |  |
| TIGECYCLINE Injection 50mg (10) |  | 43 | 180 |  |
| TMC207 100mg TABLETS (28 tabs) | 56 |  |  |  |
| TOBRAMYCIN 300mg/4mL NEBULISER SOLUTION (56) | 12 |  |  |  |
| TOBRAMYCIN Injection 40mg/1ml (10) | 6 |  |  |  |
| TOBRAMYCIN Injection 80mg/2ml (10) | 175 | 144 | 170 |  |
| TRIMETHOPRIM 200mg TABLETS (TTO) (14 TABS) |  | 21 | 10 |  |
| TRIMETHOPRIM 50mg/5ml SF SUSPENSION TTO (100ml) |  | 3 | 3 |  |
| TRIMETHOPRIM Suspension 50mg/5ml (100ml) | 2100 | 1935 | 2165 |  |
| TRIMETHOPRIM Tablets 100mg (28) | 147 | 98 | 84 |  |
| TRIMETHOPRIM Tablets 200mg (14) | 364 | 463 | 289 |  |
| TRIMETHOPRIM Tablets 200mg tto (14) | 140 | 28 |  |  |
| TRIMETHOPRIM Tablets 200mg tto (6) | 10 | 7 | 4 |  |
| VANCOMYCIN Capsules 125mg (28) | 234 | 420 | 566 |  |
| VANCOMYCIN Injection 1g (1) | 1791 | 2280 | 2123 |  |
| VANCOMYCIN Injection 500mg (1) | 530 | 589 | 599 |  |
| VORICONAZOLE Injection 200mg (1) | 99 | 94 | 333 |  |

* The hospital drug usage recording system changed in October 2014, consequently the values for October 2014 are recorded as pre-computed Defined Daily Doses per 1000 bed days grouped by antimicrobial class

| **Antimicrobial class** | **Oct-14** |
| --- | --- |
| Aminoglycosides | 114.24 |
| Beta-lactams | 1,070.48 |
| Glycopeptide antibiotics | 136.95 |
| Macrolides | 130.32 |
| Quinolones | 131.37 |
| Rifamycins | 91.59 |
| Sulfonamides & trimethoprim | 114.44 |
| Tetracyclines | 72.02 |

**Table S3.** Table showing the LCMS results for hospital and farm effluent samples (associated with each metagenome sample)

| **Antibiotic** | | **Concentration of antimicrobial present in sample (µg/litre)** | | | | | | | | | |
| --- | --- | --- | --- | --- | --- | --- | --- | --- | --- | --- | --- |
| Class | Name | AH:M:2 | AH:M:3 | AH:M:4 | AH:M:5 | AH:M:6 | DF:M:2 | DF:M:3 | DF:M:4 | DF:M:5 | DF:M:6 |
| beta-lactam | amoxicillin | < 10.0 | < 10000.0 | < 10000.0 | failed | < 10000.0 | < 1000.0 | < 50.0 | < 1000.0 | failed | < 1000.0 |
| beta-lactam | flucloxacillin | < 0.1 | < 1.0 | < 0.1 | < 0.5 | **24.5** | < 100.0 | < 1.0 | < 2.0 | < 5.0 | < 100.0 |
| beta-lactam | piperacillin | < 200.0 | < 100.0 | < 100.0 | < 200.0 | < 1000.0 | < 100.0 | < 10.0 | < 5.0 | < 10.0 | < 100.0 |
| glycopeptide | teicoplanin | < 500.0 | < 5000.0 | < 100.0 | < 1000.0 | < 100.0 | < 10000.0 | < 5000.0 | < 5000.0 | < 10000.0 | < 10000.0 |
| glycopeptide | vancomycin | **18.2** | **3160** | **29.1** | **58.1** | **90.2** | < 1000.0 | < 500.0 | < 100.0 | < 1000.0 | < 1000.0 |
| MLS | azithromycin | **57.4** | < 0.1 | failed | < 0.5 | < 10.0 | < 1000.0 | < 5.0 | failed | < 50.0 | < 1000.0 |
| MLS | clarithromycin | **13.4** | **23.6** | **17.4** | **31.1** | **7.3** | < 10.0 | < 5.0 | < 2.0 | < 10.0 | < 10.0 |
| MLS | erythromycin | < 0.1 | < 1.0 | < 1.0 | < 1.0 | < 0.1 | < 10.0 | < 1.0 | < 5.0 | < 10.0 | < 10.0 |
| phenicol | chloramphenicol | < 0.1 | < 1.0 | < 0.1 | < 1.0 | < 1.0 | < 100.0 | < 10.0 | < 10.0 | < 100.0 | < 100.0 |
| quinolone | ciprofloxacin | **59.3** | **10** | **82.9** | **10.1** | **6.5** | < 200.0 | < 100.0 | < 100.0 | < 100.0 | < 200.0 |
| quinolone | moxifloxacin | **2** | < 2.0 | < 10.0 | < 1.0 | < 20.0 | < 200.0 | < 20.0 | < 100.0 | < 100.0 | < 200.0 |
| quinolone | ofloxacin | < 0.5 | < 2.0 | < 5.0 | < 2.0 | < 0.2 | < 200.0 | < 20.0 | < 100.0 | < 50.0 | < 200.0 |
| rifamycin | rifabutin | < 0.1 | < 1.0 | < 1.0 | < 5.0 | < 0.1 | < 10.0 | < 10.0 | < 1.0 | < 50.0 | < 10.0 |
| rifamycin | rifampicin | < 0.5 | < 2.0 | < 50.0 | < 5.0 | **1.3** | < 10.0 | < 2.0 | < 50.0 | < 10.0 | < 10.0 |
| sulfonamide | sulfadiazine | < 0.1 | < 0.1 | < 0.1 | < 0.1 | < 0.1 | < 10.0 | **1.7** | **2.7** | < 10.0 | < 10.0 |
| sulfonamide | sulfamethoxazole | **257** | **1220** | < 1.0 | **173** | **15.4** | < 10.0 | < 10.0 | < 5.0 | < 10.0 | < 10.0 |
| tetracycline | demeclocycline | < 0.5 | < 100.0 | < 100.0 | < 10.0 | < 10.0 | < 100.0 | < 100.0 | < 1000.0 | < 100.0 | < 100.0 |
| tetracycline | doxycycline | < 1.0 | < 5.0 | < 1000.0 | < 5.0 | < 5.0 | < 100.0 | < 500.0 | < 1000.0 | < 50.0 | < 100.0 |
| tetracycline | tigecycline | < 1.0 | < 10000.0 | < 1000.0 | < 10.0 | < 100.0 | < 1000.0 | failed | < 1000.0 | < 100.0 | < 1000.0 |

A ‘<’ denotes a result of less than the reporting limit (listed after ‘<’), bold type indicates detected concentrations.
